# Isolation, antimicrobial resistance and virulence characterization of *Salmonella* spp. from fresh foods in retail markets in Hangzhou, China

**DOI:** 10.1371/journal.pone.0292621

**Published:** 2023-10-19

**Authors:** Min Qian, Dingting Xu, Jiankang Wang, Davood Zaeim, Jianzhong Han, Daofeng Qu

**Affiliations:** 1 School of Food Science and Biotechnology, Zhejiang Gongshang University, Hangzhou, China; 2 The Second Affiliated Hospital, School of Medicine, Zhejiang University, Hangzhou, China; 3 Agricultural Technology and Water Conservancy Service Center, Jiaxing, China; Tribhuvan University, NEPAL

## Abstract

*Salmonella* can cause severe foodborne diseases. This study investigated the prevalence of *Salmonella* spp. in fresh foods in Hangzhou market and their harborage of antibiotic resistance and virulence genes, antibiotic susceptibility, and pathogenicity. A total of 500 samples (pork, n = 140; chicken, n = 128; vegetable, n = 232) were collected over a one-year period. *Salmonella* was found in 4.2% (21) of samples with the detection rate in pork, chicken and vegetables as 4.3% (6), 6.3% (8), and 3% (7), respectively. One *Salmonella* strain was recovered from each positive sample. The isolates were identified as six serotypes, of which *S*. Enteritidis (n = 7) and *S*. Typhimurium (n = 6) were the most predominant serotypes. The majority of isolates showed resistance to tetracycline (85.7%) and/or ciprofloxacin (71.4%). Tetracycline resistance genes showed the highest prevalence (90.5%). The occurrence of resistance genes for β-lactams (*bla*_TEM-1_, 66.7%; and *bla*_SHV_, 9.5%) and aminoglycosides (*aad*A1, 47.6%; *Aac(3)-Ia*, 19%) was higher than sulfonamides (*sul*1, 42.9%) and quinolones (*par*C, 38.1%). The virulence gene *fim*A was detected in 57.1% of isolates. Gene co-occurrence analysis implied that resistance genes were associated with virulence genes. Furthermore, selected *S*. Typhimurium isolates (n = 4) carrying different resistance and virulence genes up-regulated the secretions of cytokines IL-6 and IL-8 by Caco-2 cells in different degrees, suggesting that virulence genes may play a role in inflammatory transcription. In *in vivo* virulence test, microbiological counts in mouse feces and tissues showed that all included *S*. Typhimurium were able to infect mice, with one strain showing significantly higher virulence than others. In conclusion, this study indicates *Salmonella* contamination in fresh foods in Hangzhou market poses a risk to public health and it should be closely monitored to prevent and control foodborne diseases.

## Introduction

Food safety challenged by accelerated economic globalization and trade liberalization has become a global public health issue prompting widespread concern [1]. *Salmonella* is a critical foodborne pathogen distributed globally among humans, animals, and open environments [2]. It is spread through various paths, including food and water resources, human-to-human, and human-animal contact. Recently, according to the food poisoning statistics of the National Health Insurance Administration, 50–60% of food poisoning is caused by microorganisms, among which, *Salmonella* has the highest detection rate [3–5]. *Salmonella* is a diverse gastrointestinal pathogen that can cause a variety of diseases, and its serotypes can be categorized into two main groups—typhoidal and non-typhoidal. Typhoidal serovars (TS) can cause systemic enteric fever, whereas non-typhoidal serovars (NTS) are associated with infections in a wide range of hosts, are usually zoonotic, cause acute and self-limiting gastroenteritis, and can lead to foodborne illness in humans [6, 7]. *Salmonella enterica* serotypes that cause human disease include *S*. Typhimurium and *S*. Enteritidis [8]. *Salmonella* has been frequently found in meat, especially chicken and pork. In addition, *Salmonella* infection varies with seasons, climates, years, and geographical locations [9]. *Salmonella* also is often multidrug-resistant and carries multiple virus-related genes. Drug resistance of *Salmonella* has become a significant public health concern worldwide [10]. Several reports have been recently published on *Salmonella* contamination in foods. In Shanghai, China, Ni et al. [11]found that 4.5% of lettuce sold in markets were tested positive for *Salmonella*. *Salmonella* detection rates in chicken and pork collected from Henan, China, were 45.2% and 29.2%, respectively, according to Xu et al. [12]. According to Chen et al. [13], *Salmonella* was found in 67% of pork and 46.2% of chicken samples. The majority of the studies were focused on chicken and pork products.

Antibiotics have been used for treatment, prevention, and growth promotion in animals for decades. However, their misuse has led to the selection for antibiotic-resistant bacteria (ARB) in animals and the environment [14]. ARBs can transfer resistance to bacteria infecting humans through the food chain, gene pools, phages, and DNA fragments [15]. In recent years, researchers found that Antibiotic Resistance Genes (ARGs) can be transmitted through gene levels and mobile elements with the transfer arrays like plasmid, insert sequence, transposon, integron, *etc*. Thus, ARGs in bacteria can cross the genus boundary and transmit within or between species, spreading between fungi and bacteria [16]. In addition, the pathogenicity of *Salmonella* in humans and animals is closely related to its virulence factors. *Salmonella* virulence factors include virulence islands, enterotoxins, fimbriae, and virulence plasmids [17, 18]. Most virulence genes are on virulence islands, and a few are on virulence plasmids [19]. For example, the *inv*A gene is located on the virulence island and is involved in host recognition and invasion of intestinal mucosal epithelial cells [18]. The *spv*C gene is mainly located on the virulence plasmid and plays a role in the intracellular proliferation and survival of *Salmonella* within the host [17]. Caco-2 cells are an intestinal epithelial model widely used to study the organization and function of human intestinal cells *in vitro* [20]. *Salmonella* can adhere to and invade Caco-2 cells and induce the expression and upregulation of pro-inflammatory cytokines in the cells [21]. IL-6 regulates cell growth and differentiation, regulates immune responses, and is also involved in acute phase responses and hematopoiesis [22]. IL-8 is the major pro-inflammatory CXC chemokine secreted by intestinal epithelial cells in response to bacterial entry [23]. Studies have shown that the amount of intracellular bacteria in the Caco-2 cell line increases with increased inflammation during infection [24]. However, there is no detailed report on whether virulence genes carried by bacteria affect cytokine expression and whether the association between antibiotic resistance and virulence increases pathogenic risk.

In this study, samples of fresh food were collected from the retail markets in Hangzhou, China over a one-year period and detected for *Salmonella*. The recovered *Salmonella* isolates were determined for antibiotic resistance, virulence and presence of antibiotic resistance and virulence associated genes. The correlation between antibiotic resistance genes and virulence genes was analyzed.

## Materials and methods

### Sampling

From March 2020 to February 2021, 500 samples (140 raw minced pork, 128 raw chicken carcasses, and 232 vegetables including cilantro, lettuce, tomatoes, cucumbers, spinach and cabbage) were randomly collected from retail markets in Hangzhou, Zhejiang Province, China, and placed in sterile sampling bags/tubes. All samples were stored at 2–8°C and transported to the laboratory for processing within 24 hours.

### Detection and isolation of *Salmonella*

*Salmonella* was detected using the method described previously [25]. Briefly, 25 g sample was added to 225 ml of buffered peptone water (BPW; HB4084, Hopebio, China). All samples were incubated at 37°C for 18 h for pre-enrichment. One mL of the pre-enrichment solution was transferred to 9 ml of selenite cysteine broth (SC; HB4085, Hopebio, China) and incubated at 37°C for 24 h for enrichment. SC broth cultures were subsequently taken and streaked onto XLD plates. The plates were incubated at 37°C for 24–48 h. Suspected *Salmonella* spp. colonies (red colonies with black centers) on the XLD plates were picked for subsequent experiments. *Salmonella* was initially screened by biochemical assays, namely indole, sugar fermentation assay, production of urease and H_2_S, and production of lysine and ornithine decarboxylase. Genomic DNA of *Salmonella* species was extracted by the boiling method described by Coo et al. [26] to be used as template DNA for PCR. The isolates were identified as *Salmonella* by PCR detection of the specific gene *inv*A, as previously described [27]. A 25 μl of reaction mixture was prepared, containing 12.5 μl 2X Taq PCR Master Mix (manufacture information), 0.5 μl each primer (10 μM), 1.0 μl template DNA, and 10.5 μl ddH_2_O. PCR condition was as follows: pre-denaturation at 96°C for 5 min; 30 cycles of denaturation at 95°C for 30 s, annealing at 50°C for 90 s, and extension at 72°C for 60 s; extension at 72°C for 5 min; incubation at 12°C. After the program, the PCR product was stored at 4°C. Five μl of PCR amplification product was electrophoresed in a 1.5% agarose gel, visualized, and recorded in Gel Doc XR (Bio-Rad, USA).

### Serotyping

The serotypes of the isolated *Salmonella* were identified by slide agglutination method, and the O antigen and H antigen types were determined, respectively. According to the product manual of *Salmonella* diagnostic serum and antigenic formulae of the salmonella serovars, the serotype of *Salmonella* was determined based on the antigenic formula [28].

### Antibiotic susceptibility testing

Kirby Bauer disk diffusion method for antimicrobial susceptibility testing was performed on all isolates according to the Clinical and Laboratory Standards Institute guidelines [29]. The 12 antimicrobials tested included ciprofloxacin (5 μg), gentamicin (10 μg), streptomycin (10 μg), erythromycin (15 μg), ampicillin (10 μg), amoxicillin (25 μg), enrofloxacin (5 μg), kanamycin (30 μg), cefotaxime (30 μg), sulfamethoxazole (30 μg), cephalothin (30 μg), tetracycline (30 μg). Antibiotic disks were from Hangzhou Microbial Reagent Company (Zhejiang, China). Inoculated plates were incubated at 37°C for 24 h. *Salmonella* strains were assessed based on the size of the inhibition zone.

### Detection and correlation analysis of ARGs and VGs

All *Salmonella* isolates were tested for antimicrobial resistance genes, including quinolones (*par*C), aminoglycosides (*aad*A1 and *Aac(3)-Ia*), sulfonamides (*sul*1), β -lactamase (*bla*_TEM-1_ and *bla*_SHV_), tetracycline (*tet*(A) and *tet*(B)) by PCR. The flagellin *fli*C gene, fimbrial toxin *fim*A gene, enterotoxin *stn* gene, and virulence plasmid *spv*C gene related to *Salmonella* pathogenicity were detected. The DNA extraction steps were as described above. PCR reagent concentrations and cycling conditions (including annealing temperature) were consistent with previous studies [30, 31]. Primers are shown in S1 Table.

Co-occurrence and contributor networks of ARGs and VGs were analyzed by R software (X64 3.5.1) with the package of "psych" and "vegan" based on Spearman’s rank correlations (P < 0.05 indicating a statistical significance) and visualized by Gephi.

### *In vitro* virulence test

#### Caco-2 cell maintenance and preparation

Caco-2 cells are a human colonic epithelial cell line (ATCC, BFB, Shanghai). Cells were seeded into plastic Petri dishes. Cells were cultured in Dulbecco’s Modified Eagle Medium (DMEM, Genomcell, China) supplemented with 10% fetal calf serum (FBS, Genomcell, China) and 1% antibiotics (penicillin/streptomycin, Genomcell) at 37°C. Once the cells reached 90% confluence, cells were trypsinized (0.05%, Genomcell, China) and seeded at the desired density in 96-well tissue culture plates (Nunc, France) containing 200 μL of complete medium per well. Cells were incubated at 37°C for at least 24 h after seeding in a humidified atmosphere containing 5% CO_2_ to achieve complete confluency.

#### Determination of Caco-2 invasion by *Salmonella*

The 96-well plate monolayer was rinsed twice with phosphate-buffered saline (PBS, pH 7.4). A strain of *S*. Typhimurium, which carried the highest number of types and drug resistance and virulence genes from each season was selected (spring, *par*C, *sul*1; summer, *tet*(A), *bla*_TEM-1_, *fim*A; autumn, *aad*A1, *spv*C; winter, *aad*A1). The suspension of different *Salmonella* strains was added to washed Caco-2 cells at an initial concentration of 10^6^ CFU/mL. The plates were incubated at 37°C for 4 h in a 5% CO_2_ incubator. They were maintained for 1 h in DMEM (Genomcell, China) containing 50 μg/mL gentamicin per well to inactivate extracellular bacteria. After incubation, the infected cells were lysed by adding 1% Triton X-100 (Sigma). Lysates were plated on LB plates and incubated at 37°C for 24 h. The invasion rate was calculated as the percentage of invading bacteria versus initial bacterial addition.

#### Total RNA isolation, cDNA synthesis, and real-time PCR

Caco-2 cell maintenance and preparation and *Salmonella* infection of Caco-2 cells were performed as described above. According to the manufacturer’s instructions, total RNA was extracted from cells using RNAiso Plus (Takara, Japan). The RNA was reverse transcribed with a HiScript II Q RT SuperMix for qPCR (Vazyme, China). The resulting cDNA was quantified by RT-PCR using AceQ qPCR SYBR Green Master Mix (Vazyme, China). The RT-PCR reaction mixture contained 2 μl cDNA, 0.8 μl of different primers (concentration of primers), and 10 μl SYBR Green Master Mix, with a total volume of 20μl. The reaction program was as follows: 15 min at 95°C; followed by 40 cycles of 5 s at 95°C, 30 s at 60°C and 30 s at 72°C in 96-well plates with the ABI 9700HT Sequence Detection System (Applied Biosystems, USA). Using the human GAPDH gene as an internal reference, the relative expression of each related gene mRNA was calculated by the 2^-ΔΔCt^ method. Primers are shown in S1 Table.

### *In vivo* virulence test

#### Infection model

Six-week-old male mice were provided by Ziyuan Laboratory Animal Technology Co., Ltd (Hangzhou, China), free of pathogens and weighing 20–25 g. Before testing, mice were kept in a controlled environment with a 12 h light, 12 h dark cycle at 22°C, with food and water provided ad libitum. A total of 30 mice were divided into 5 groups, one group was given only sterile saline solution (0.9% w/v) and 4 experimental groups were inoculated with the target strain. Mice were fasted for 12 h before inoculation and subsequently, 1 ml of bacterial suspension (1x10^8^ CFU/ml) was administered by the oral needle. After inoculation (day 0), food and water were provided ad libitum. On day 5, the mice were executed by breaking their necks. Immediately after the execution, the ileum, liver, and spleen were removed and microbiological analysis was performed. The breeding and use of mice were supervised by the Laboratory Animal Welfare and Ethics Committee of Hangzhou Normal University under the ethical approval number HSD20211201.

#### Clinical observations

Animal weights were measured every 24 h. To lessen the initial weight change for each animal, the baseline weight was recorded on day 0 and the value was deducted from the weight on the following days.

#### Microbiological analyses

Feces samples, liver, spleen, and ileal tissues were mixed with sterile saline-peptone solution (w/v, 1:10) and homogenized. A proportion of 0.2 ml of the dilution was applied to bright green agar (BGA) plates. Plates were incubated at 37°C for 24 h for bacterial counting.

### Statistical analysis

Statistical analysis was conducted using SPSS 26.0. The differences between groups/strains were assessed by Chi-square test/ANOVA with p < 0.05 regarded as significant difference. Data was visualized using Origin 2021.

## Results

### Prevalence and serotypes of *Salmonella*

*Salmonella* contamination in different fresh foods collected at different seasons was investigated. A percentage of 4.2% samples (21/500) was found to have *Salmonella* (Table 1). One *Salmonella* isolate was recovered from each positive sample. The difference of *Salmonella* detection rate was not statistically different among food types or seasons. However, both pork (4.3%, 6/140) and chicken (6.3%, 8/128) showed numerically higher detection rate of *Salmonella* compared to vegetables (3%, 7/232). The detection rate of *Salmonella* in summer (5.9%, 8/135) was numerically higher than that in other seasons (spring, 4.2%, 5/120, autumn, 3.9%, 5/130, winter, 3/115).

**Table 1:** The prevalence of *Salmonella*.

	Pork	Chicken	Vegetable	Total
Positive number	6/140	8/128	7/232	21/500
Positive rate (%)	4.3	6.3	3.0	4.2
Season	Month	Total number	Positive number (%)	
Spring	2–5	120	5 (4.2)	
Summer	6–8	135	8 (5.9)	
Autumn	9–11	130	5 (3.9)	
Winter	12–2	115	3 (2.6)	

**Fig 1:**
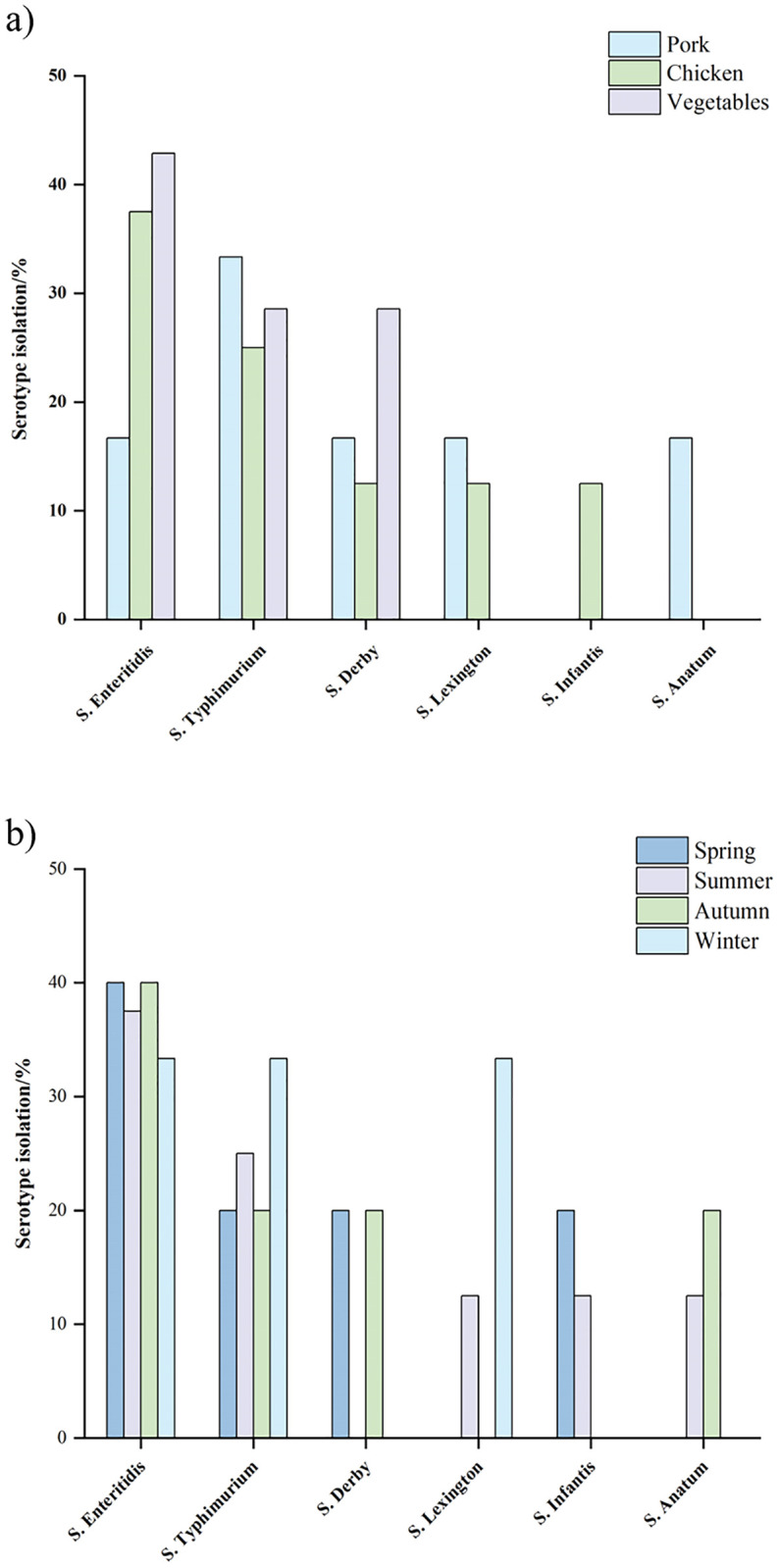
Serotype distribution of the *Salmonella* isolates. a) Fresh foods. b) Seasons.

The distribution of *Salmonella* serotypes from different seasons, fresh meat, and vegetables is shown in Fig 1. Among 21 *Salmonella* isolates, 6 *Salmonella* serotypes were identified. The most predominant serotype was *S*. Enteritidis (n = 7, 33.3%), followed by *S*. Typhimurium (6, 28.6%), *S*. Derby (4, 19%), *S*. Lexington (2, 9.5%), *S*. Infantis (1, 4.8%), and *S*. Anatum (1, 4.8%). Of the 6 different *Salmonella* serotypes, 5, 5 and 3 types were isolated from pork, chicken and vegetables (Fig 1a), and 4, 5, 4 and 3 types were recovered from spring, summer, autumn and winter, respectively (Fig 1b).

The antibacterial susceptibility of 21 *Salmonella* strains is shown in Table 2. The strains showed varying degrees of resistance to antimicrobial drugs. The largest proportion of *Salmonella* showed resistance to tetracycline (85.7%) followed by ciprofloxacin (71.4%), streptomycin (66.7%), erythromycin (66.7%), gentamycin (52.4%), ampicillin (38.1%), enrofloxacin (33.3%), cephalothin (28.6%), cefotaxime (23.8%), sulfamethoxazole (19.1%), amoxicillin (19.1%) and kanamycin (19.1%).

**Table 2:** *Salmonella* antibiotic resistance.

Antibiotic	Pork (n = 6) (%)	Chicken (n = 8) (%)	Vegetable (n = 7) (%)	Spring (n = 5) (%)	Summer (n = 8) (%)	Autumn (n = 5) (%)	Winter (n = 3) (%)	Total (%)
Ciprofloxacin	5 (83.3)	7 (87.5)	3 (42.9)	3 (60)	7 (87.5)	3 (60)	2 (66. 7)	15 (71.4)
Gentamycin	4 (66. 7)	5 (62.5)	2 (28.6)	2 (40)	5 (62.5)	3 (60)	1 (33.3)	11 (52.4)
Streptomycin	2 (33. 3)	6 (75)	6 (85.7)	3 (60)	6 (75)	3 (60)	2 (66. 7)	14 (66.7)
Erythromycin	4 (66. 7)	4 (50)	6 (85.7)	3 (60)	6 (75)	2 (40)	3 (100)	14 (66.7)
Ampicillin	3 (50)	2 (25)	3 (42.9)	1 (20)	4 (50)	1 (20)	2 (66.7)	8 (38.1)
Amoxicillin	2 (33. 3)	2 (25)	/	1 (20)	2 (25)	/	1 (33.3)	4 (28.6)
Enrofloxacin	1 (16. 7)	4 (50)	2 (28.6)	2 (40)	3 (37.5)	1 (20)	1 (33.3)	7 (33.3)
Kanamycin	2 (33. 3)	1 (12.5)	1 (14.3)	1 (20)	2 (25)	1 (20)	/	4 (19.1)
Cefotaxime	2 (33. 3)	3 (37.5)	/	1 (20)	2 (25)	1 (20)	1 (33.3)	5 (23.8)
Sulfamethoxazole	2 (33. 3)	1 (12.5)	1 (14.3)	/	2 (25)	1 (20)	1 (33.3)	4 (19.1)
Cephalothin	/	3 (37.5)	3 (42.9)	1 (20)	3 (37.5)	2 (40)	/	6 (28.6)
Tetracycline	4 (66.7)	7 (87.5)	7 (100)	4 (80)	7 (100)	4 (80)	3 (100)	18 (85.7)

### PCR detection of ARGs and VGs

**Fig 2:**
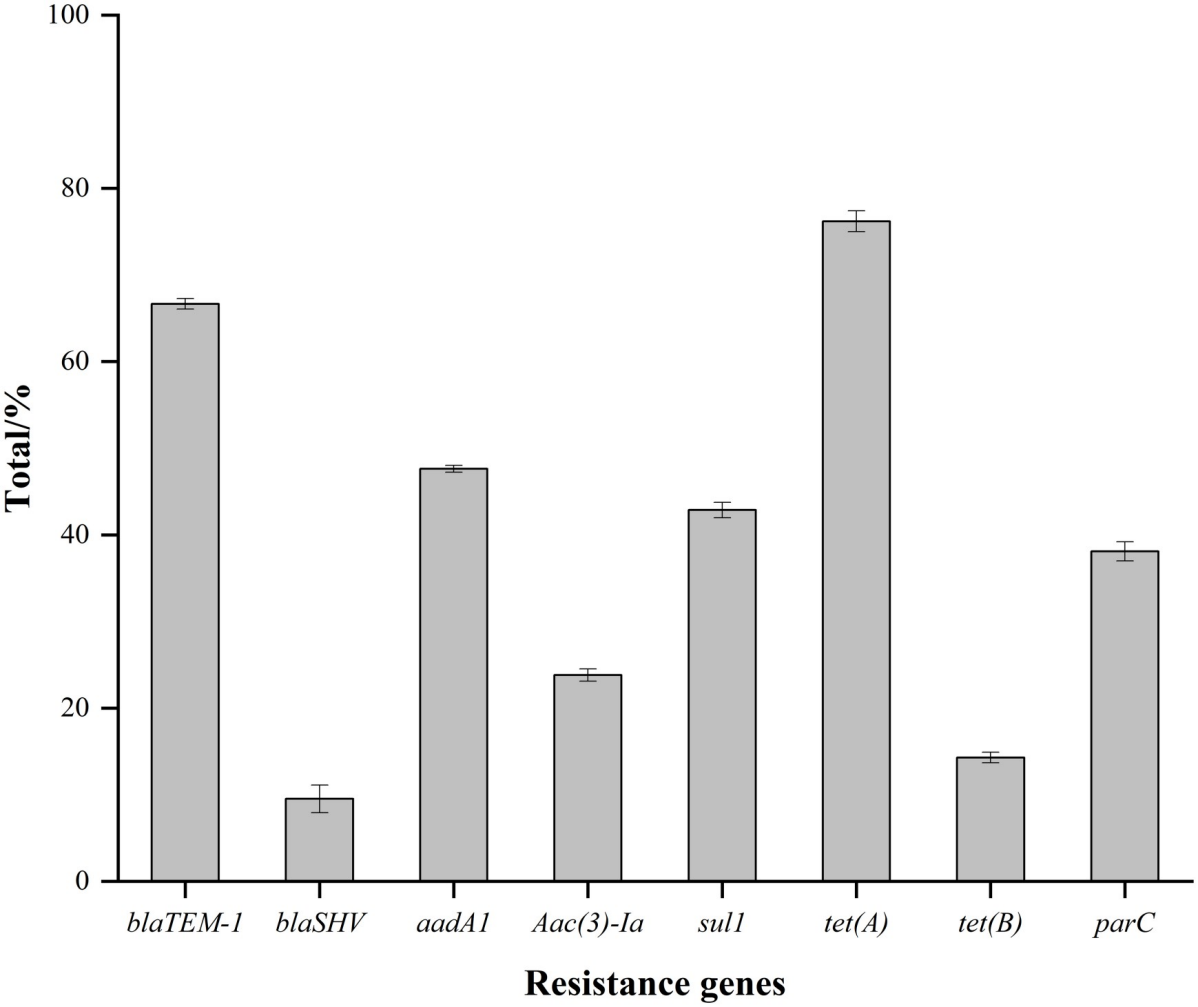
The detection rate of resistance genes. Error bars correspond to the standard deviation of the means and letters indicate statistically significant differences between groups.

The resistance genes for tetracycline exhibited the highest detection rate (90.5%) in 21 *Salmonella*, which was consistent with the phenotypic characterization of antimicrobial resistance (Table 3). Resistance genes were observed to be higher for β-lactams and aminoglycosides than sulfonamides and quinolones (Fig 2).

**Table 3:** Antimicrobial resistance genes of the *Salmonella* isolates.

Classification	Resistance gene	Pork (n = 6) (%)	Chicken (n = 8) (%)	Vegetable (n = 7) (%)	Spring (n = 5) (%)	Summer (n = 8) (%)	Autumn (n = 5) (%)	Winter (n = 3) (%)	Total (%)
β-lactams	*bla*_TEM-1_	5 (83.3)	6 (75)	2 (28.6)	2 (40)	6 (75)	3 (60)	2 (66.7)	13 (66.7)
	*bla*_SHV_	1 (16.7)	1 (12.5)	/	/	1 (12.5)	/	1 (33.3)	2 (9.5)
Aminoglycosides	*aad*A1	2 (33.3)	6 (75)	2 (28.6)	2 (40)	5 (62.5)	2 (400)	1 (33.3)	10 (47.6)
	*Aac(3)-Ia*	1 (14.3)	2 (25)	1 (14.3)	/	3 (37.5)	1 (20)	/	4 (19)
Sulfa	*sul*1	5 (83.3)	2 (25)	2 (28.6)	/	4 (50)	3 (60)	2 (66. 7)	9 (42.9)
Tetracyclines	*tet*(A)	4 (66.7)	6 (75)	6 (85.7)	3 (60)	7 (87.5)	3 (60)	3 (100)	16 (76.2)
	*tet*(B)	/	2 (25)	1 (14.3)	/	1 (12.5)	2 (40)	/	3 (14.3)
Quinolones	*par*C	4 (66.7)	4 (50)	/	2 (40)	4 (50)	2 (40)	/	8 (38.1)

The virulence genes in *Salmonella* isolates are shown in Table 4. *fim*A was detected at the highest rate of 57.1%, followed by *fli*C (33.3%), *spv*C (23.8%), and *stn* (19.1%).

**Table 4:** Virulence genes of *Salmonella* isolates.

Virulence genes	Pork (n = 6) (%)	Chicken (n = 8) (%)	Vegetable (n = 7) (%)	Spring (n = 5) (%)	Summer (n = 8) (%)	Autumn (n = 5) (%)	Winter (n = 3) (%)	Total (%)
*fli*C	2 (33. 3)	4 (50)	1 (14.3)	2 (40)	3 (37.5)	1 (20)	1 (33. 3)	7 (33. 3)
*fim*A	3 (50)	6 (75)	3 (42.9)	3 (60)	5 (62.5)	3 (60)	1 (33.3)	12 (57.1)
*stn*	1 (16.7)	3 (37.5)	/	/	2 (25)	1 (20)	1 (33.3)	4 (19.1)
*spv*C	2 (33. 3)	3 (37.5)	1 (14.3)	1 (20)	3 (37.5)	1 (20)	/	5 (23.8)

### Co-occurrence analysis of ARGs and VGs

**Fig 3:**
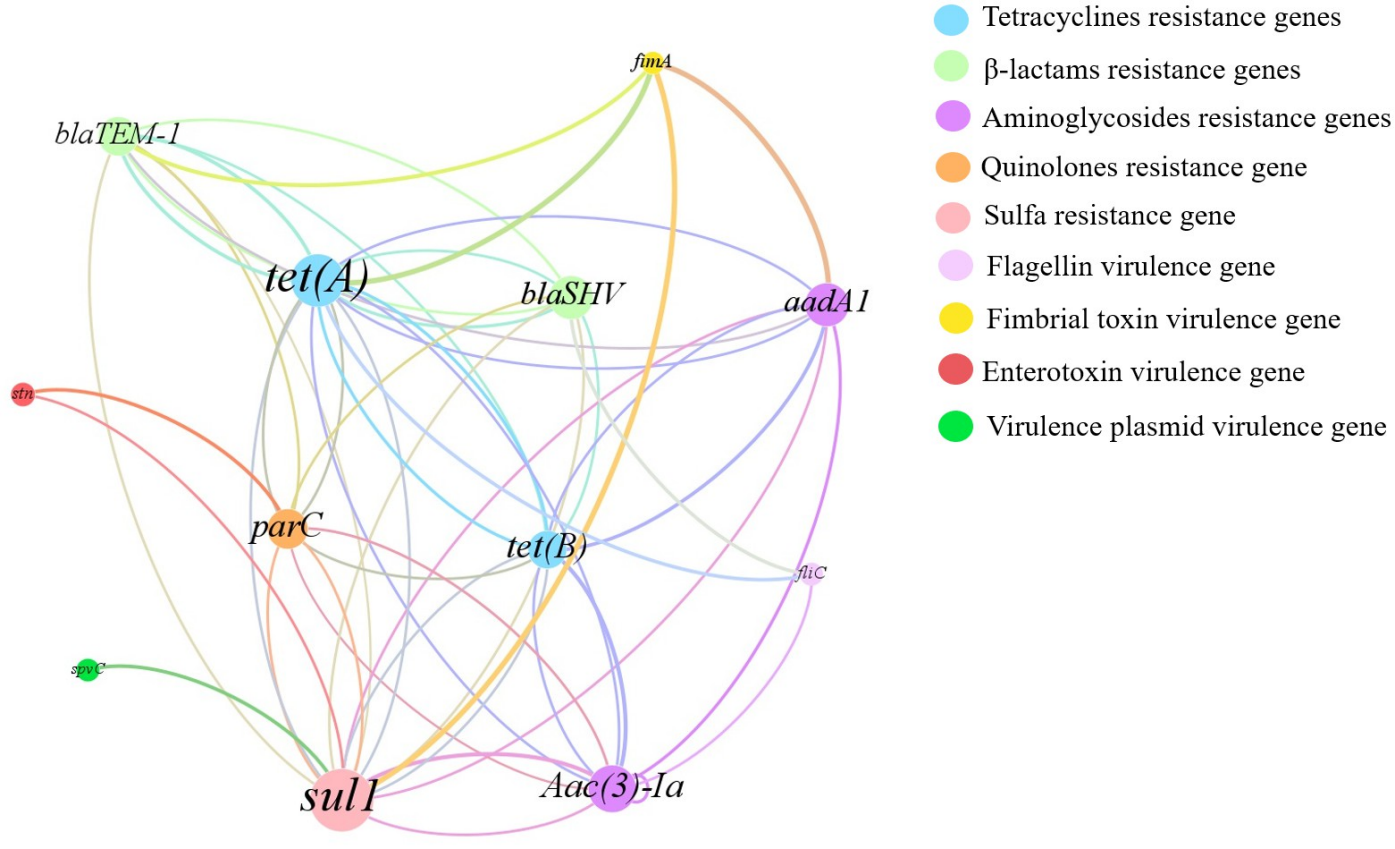
Co-occurrence gene network of ARG subtypes and VGs in MDR isolates collected. **9 different colors represent 5 kinds of antibiotics and 4 virulence genes**. Nodes belonging to the same kinds of VGs or resistant to the same class of antibiotics are presented in the same color. A connection represents a significant (P<0.05) correlation. The size of each node is proportional to the number of connections.

As shown in Fig 3, the gene co-occurrence analysis of the ARGs network showed a close correlation between ARGs and VGs (with a correlation of P < 0.05). In the figure, resistance genes were significantly associated with the virulence gene *fim*A. The *fim*A gene was mainly co-occurring with *sul*1, *aad*A1, *tet*(A) and *bla*_TEM-1_ genes for resistance to sulfonamides, β-lactams, tetracyclines and aminoglycosides, respectively. In addition, *fli*C co-occurred mainly with *bla*_SHV_, *Aaca(3)-I* and *tet*(A). Notably, all these resistance genes were detected at relatively high levels, especially *sul*1, *aad*A1, and *tet*(A), which were significantly associated with *fim*A. In addition, *bla*_SHV_ and *par*C genes, which have relatively low detection rates, were also associated with VGs such as *fli*C and *stn*. In addition, the network showed the complexity of the linkage between ARGs of tetracyclines, β-lactams, sulfonamides, aminoglycosides, and quinolones (*tet*(A), *tet*(B), *bla*_TEM-1_, *bla*_SHV_, *sul*1, *aad*A1, *Aaca(3)-I*, *par*C, etc.).

### *In vitro* virulence

**Fig 4:**
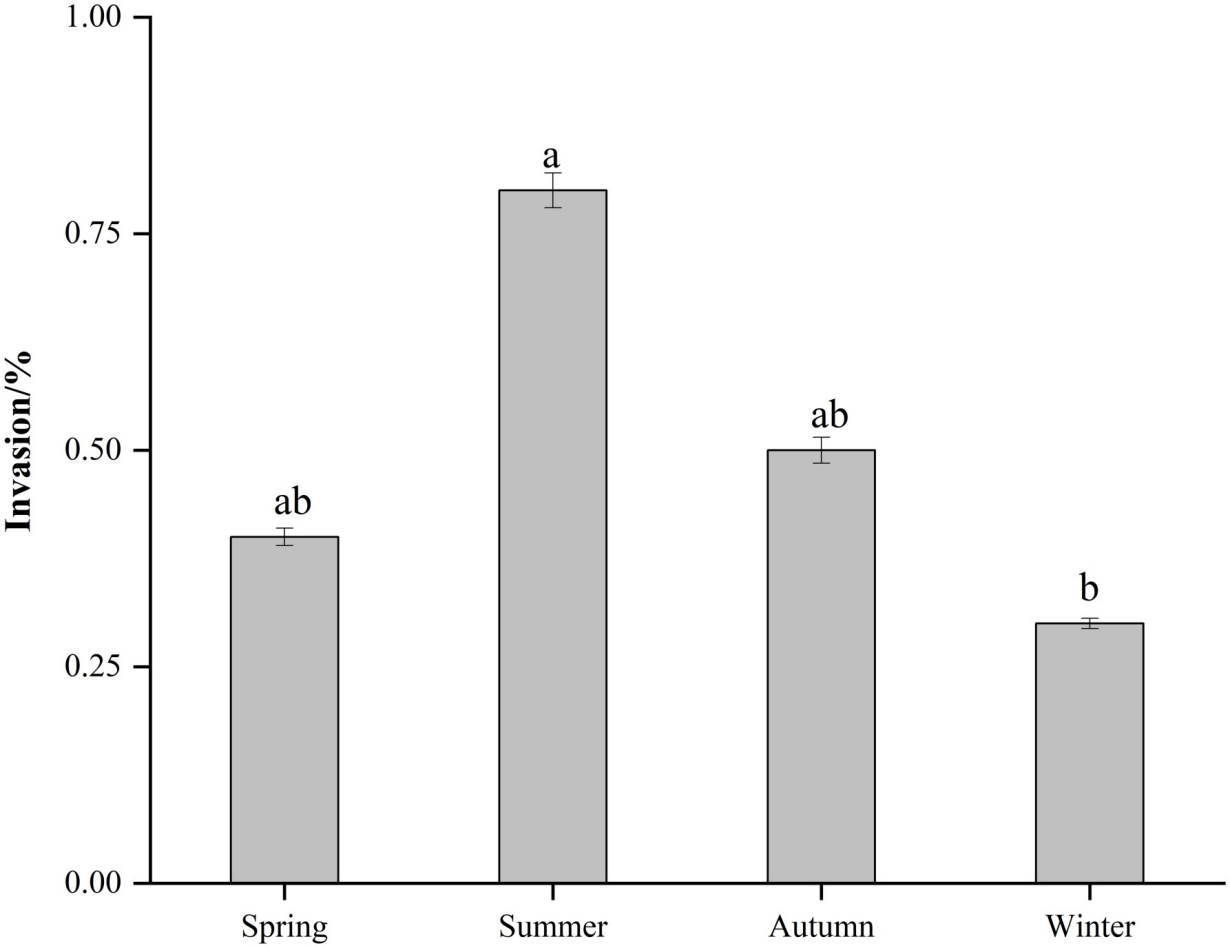
Percentage of invasion of Caco-2 cells by *Salmonella* strain. Error bars correspond to the standard deviation of the means and letters indicate statistically significant differences between groups.

The invasive capacity ranged from 0.3% to 0.8% by spring, summer, autumn, and winter strains of *S*. Typhimurium. The ability to invade Caco-2 cells was the lowest for winter strain and the highest for summer strain (Fig 4).

**Fig 5:**
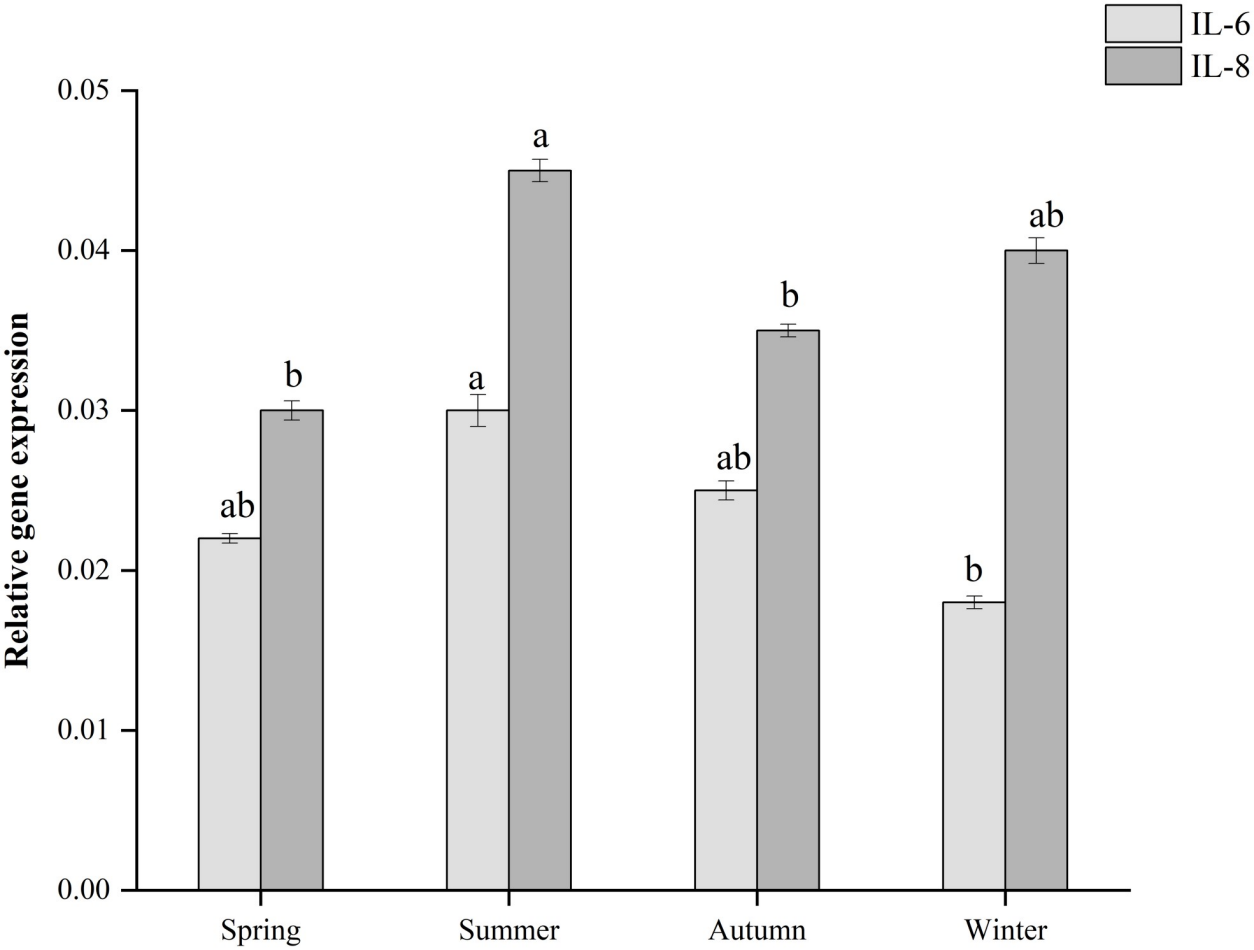
Expression of IL-6 and IL-8 mRNA was determined by real-time qPCR and normalized to GAPDH using the 2^-ΔΔCt^ method. Bars represent the mean of triplicates. Error bars correspond to the standard deviation of the means and letters indicate statistically significant differences between groups.

Cytokine (IL-6, IL-8) production by *Salmonella*-infected Caco-2 cells was studied. As shown in Fig 5, *Salmonella* in samples from different seasons could induce inflammatory transcription in Caco-2 cells. The highest mRNA expression of IL-6 and IL-8 was observed in *Salmonella*-infected Caco-2 cells from summer strain. The least mRNA expression of IL-6 was observed in *Salmonella*-infected Caco-2 cells from winter strain (P<0.05). The data suggest that Caco-2 cells infected with *Salmonella* carrying different virulence genes may induce different levels of IL-6 and IL-8 secretion.

### *In vivo* virulence

**Fig 6:**
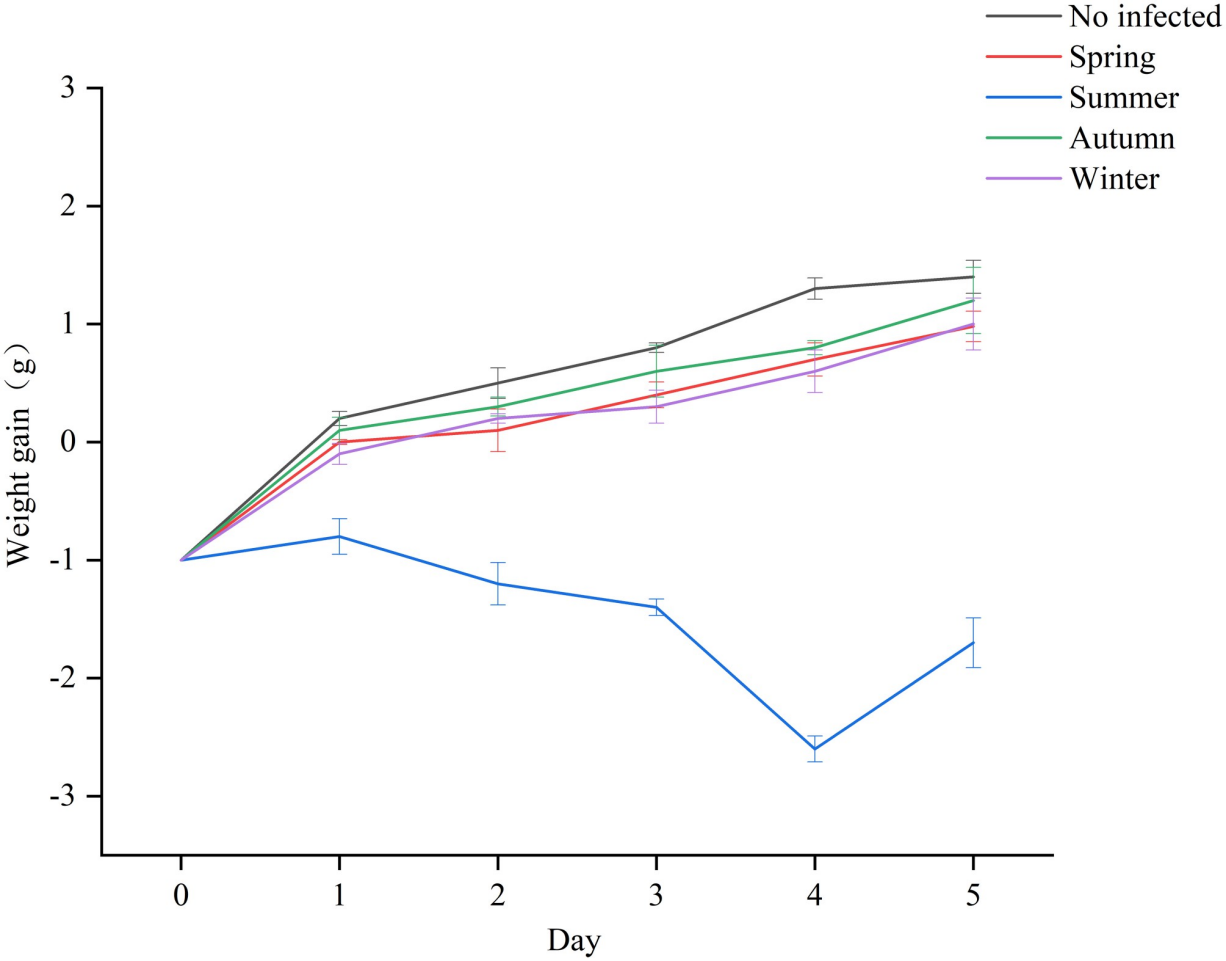
Body weight observation of mice infected with *Salmonella* strains. Data represent the mean of 6 mice per group. The means of weight in each group were statistically different (P<0.05).

For body weight, on day 0, all mice lost an average of 1 g of body weight after fasting. The control group began to show rapid weight recovery on day 1 after the start of the experiment and continued to gain weight (Fig 6). The group of mice infected with spring, autumn, and winter strains of *Salmonella* performed similarly to the control group, with an increase in body weight on the fourth and fifth day after infection. In contrast, mice inoculated with *Salmonella* summer samples showed significant weight loss on all monitoring days, more severe on the fourth day with a value of -2.6 g (P<0.05).

**Fig 7:**
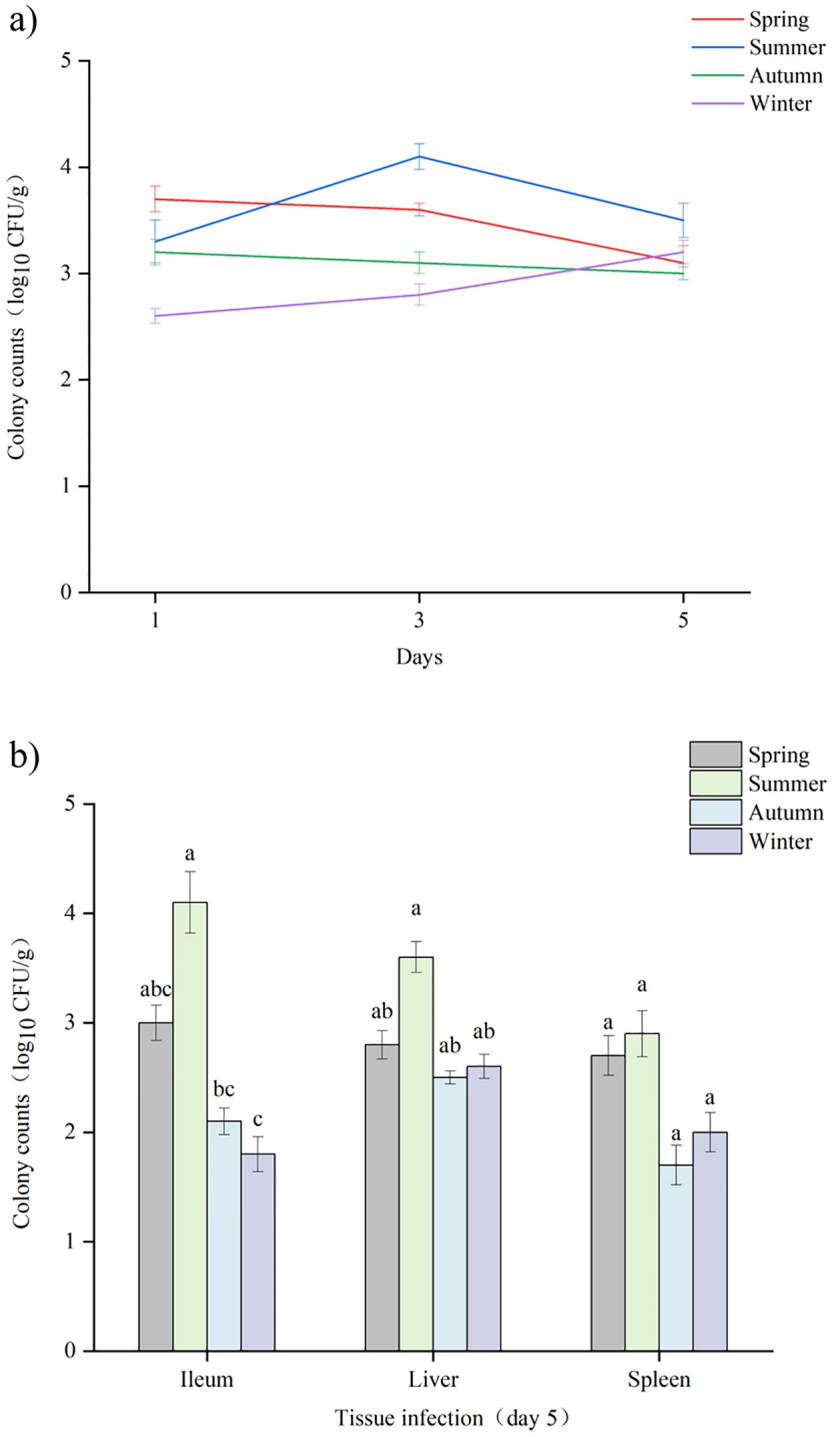
Microbiological analysis of feces and tissues. a) Colony counts in feces of infected mice (log_10_CFU/g). The mean number of feces in each group was statistically different (P< 0.05). b) Colony counts in tissues of infected mice (log_10_CFU/g). Means in each tissue were statistically different (P< 0.05).

The uninfected control group did not show the presence of *Salmonella* in the feces. The number of bacteria was found in all groups, with the highest amounts on day 3 and 5 (Fig 7a). On the first day after infection, more CFU was found in the feces of mice infected with the spring strain than that in the other groups (P<0.05). On day 3, the group infected with summer samples had more CFU, and on day 5, there was little difference between the mice of the different infected groups (P<0.05). Regarding the microbiological analysis of the ileum, liver, and spleen, the presence of *Salmonella* was observed in all three tissues of the infected animal groups (Fig 7b). The tissues of the *Salmonella* group infected with the summer sample showed the highest number of bacteria compared to the mice infected with the other sample strains (P<0.05).

## Discussion

With rapid economic development, people’s awareness of food safety has gradually increased. Foodborne diseases and poisoning are mainly due to foodborne pathogenic bacteria [1]. *Salmonella* is a common cause of food poisoning worldwide, leading to epidemics in birds, reptiles, amphibians, and insects. Vegetable, fruit, and egg products are susceptible to *Salmonella* contamination in the production, processing, and marketing of such products, posing food safety concerns [32, 33]. Therefore, it is necessary to monitor the prevalence of *Salmonella*. The current study showed that the detection rate of *Salmonella* in different fresh foods reached 4.2%, and the detection rate in meat samples (5.2%) was slightly higher than that in vegetable samples (3.1%), which was consistent with the previous report by Li et al. [34], indicating that meat samples tend to be more susceptible to *Salmonella* contamination than vegetables. In this study, it was hypothesized that the rate of *Salmonella* contamination would be influenced by the season. This study found the detection rate of *Salmonella* was the highest in summer, and similar results have been reported in the relevant literature. In summer (June-August), both higher temperature and humidity in Hangzhou may provide a breeding ground for *Salmonella*. We identified 6 serotypes from 21 *Salmonella* strains, among which *S*. Enteritidis and *S*. Typhimurium were the most prevalent. Zhang et al. [35] found that among the clinically-isolated *Salmonella* from Shanghai, a city geographically close to Hangzhou, the two most abundant serotypes were also *S*. Typhimurium and *S*. Enteritidis.

Previous studies have shown that *Salmonella* is multidrug-resistant [36]. In this study, 21 *Salmonella* isolates were resistant to at least one antibacterial agent, and most isolates exhibited multi-resistance, mainly to tetracycline, ciprofloxacin, streptomycin, and erythromycin. According to previous reports [37, 38], *Salmonella* strains were mostly resistant to streptomycin, tetracyclines, and quinolones, which is consistent with our findings. Resistance genes for tetracyclines showed the highest detection rate, which was consistent with the resistance phenotype of *Salmonella* isolates. These data suggest that resistance to specific antimicrobial agents may be related to antimicrobial resistance genes.

The pathogenicity of *Salmonella* is related to the interaction between antibiotic and virulence factors [39]. Infections caused by antibiotic-resistant *Salmonella* with virulence genes have taken a long time to recover [40]. *Salmonella fimbriae* virulence gene *fim*A contributes to epithelial cell colonization [41]. In this study, the *fim*A virulence gene had a high detection rate and when tested with an invasion assay using intestinal epithelial (Caco-2) cells, they showed varying degrees of invasiveness, which is consistent with previously reported results [42]. In the current study, the highest *fim*A detection rate was found in both meat, vegetable, and different seasonal samples. The flagellin *fli*C gene is associated with biofilm formation, and *Salmonella* significantly enhances resistance to external pressure after biofilm formation [43]. *Salmonella* enterotoxin gene *stn* is a significant factor in gastroenteritis [44]. *Salmonella* virulence plasmid *spv*C is essential for systemic virulence [45]. The correlation between virulence factors and antibiotic resistance has been studied worldwide [46]. In the current study, the two genes with the highest detection rate, *tet*(A) and *sul*1 coexisted significantly with *fim*A. The virulence gene *fim*A mostly co-occurred with a total of 4 resistance genes (*aad*A1, *sul*1, *tet*(A), *bla*_TEM-1_), which were more than other virulence genes elements. After *Salmonella* invaded Caco-2, the summer samples had a very high invasion rate (0.8%), and their invasion ability was significantly higher than other samples (P<0.05). In addition, the mRNA levels of inflammatory factors IL-6 and IL-8 increased. The results showed that the mRNA expression of IL-6 and IL-8 was the highest in Caco-2 cells infected with *Salmonella* in summer strain and the least in winter strain, which was consistent with the detection rates of antibiotic resistance genes and virulence genes carried by *Salmonella* samples in different seasons. It is speculated that virulence genes may affect inflammatory transcription in the Caco-2 cell line. Evidence from *in vivo* experiments indicates that *Salmonella* infection of mice in summer exhibits significantly higher virulence effects than in other seasons, which is consistent with the results of *in vitro* experiments. In addition to this, when antibiotic resistance and virulence correlations are considered, isolates with strong antibiotic resistance and high virulence levels are at the highest risk. The combination of antibiotic resistance and virulence poses a major and alarming problem for food safety and public health. The mechanism behind the coupling is worth investigating.

## Conclusion

In conclusion, this study showed that *Salmonella* contamination rates vary by season and source in different fresh foods, that antibiotic-resistant *Salmonella* isolates existed, and that isolates containing virulence genes exhibited virulence effects *in vitro* and *in vivo*, in addition to the combination of antibiotic resistance and virulence increasing the risk of *Salmonella* pathogenicity. This may pose potential risks to food safety and human health. Therefore, the fresh food market requires strict hygiene standards in different seasons to reduce the occurrence of *Salmonella* contamination, antibiotic resistance, and virulence characteristics. They are critical parameters in improving food safety, reducing the risk of foodborne illness, and selecting effective antibiotics to treat salmonellosis.

## Supporting information

S1 Table Gene primer information. (XLSX)
